# Electroosmosis-Optimized Thermal Model for Peristaltic Transportation of Thermally Radiative Magnetized Liquid with Nonlinear Convection

**DOI:** 10.3390/e24040530

**Published:** 2022-04-10

**Authors:** Yasir Akbar, Hammad Alotaibi

**Affiliations:** 1 Department of Mathematics, COMSATS University Islamabad, Islamabad 45550, Pakistan; 2 Department of Mathematics and Statistics, College of Science, Taif University, P.O. Box 11099, Taif 21944, Saudi Arabia; hm.alotaibi@tu.edu.sa

**Keywords:** nonlinear convection, variable viscosity, heat transfer, entropy production, wavy fluid flow, electroosmosis

## Abstract

The present study addresses the heat transfer efficiency and entropy production of electrically conducting kerosene-based liquid led by the combined impact of electroosmosis and peristalsis mechanisms. Effects of nonlinear mixed convection heat transfer, temperature-dependent viscosity, radiative heat flux, electric and magnetic fields, porous medium, heat sink/source, viscous dissipation, and Joule heating are presented. The Debye–Huckel linearization approximation is employed in the electrohydrodynamic problem. Mathematical modeling is conducted within the limitations of *δ* << 1 and *Re* → 0. Coupled differential equations after implementing a lubrication approach are numerically solved. The essential characteristics of the production of entropy, the factors influencing it, and the characteristics of heat and fluid in relation to various physical parameters are graphically evaluated by assigning them a growing list of numeric values. This analysis reveals that heat transfer enhances by enhancing nonlinear convection and Joule heating parameters. The irreversibility analysis ensures that the minimization of entropy generation is observed when the parameters of viscosity and radiation are held under control. Fluid velocity can be regulated by adjusting the Helmholtz–Smoluchowski velocity and magnetic field strength.

## 1. Introduction

Mixed convection heat transfer describes the heat transmission mode in which both natural convection and forced convection effects cannot be disregarded. It has a sophisticated thermal transfer feature, distinct from the characteristics of natural or forced convection, due to variations in the thermophysical characteristics of the liquid, also associated with the impact of buoyancy. The relationship between buoyancy and shear/lateral force complicates the task. For the case of mixed convection of the auxiliary flow, wherein flow and buoyancy direction are the same, the rate of heat evacuation across a heated surface can be lowered and may affect the performance and protection of numerous industrial appliances [1]. Mixed convection heat transfer studies have been conducted in the nuclear power sector to analyze the work of safety systems in nuclear power plants running at elevated temperatures in order to prepare for urgent operating scenarios [2,3]. Apart from that, mixed convection also occurs in supercritical solar collectors [4], rotating shafts, small modular reactors [5], water-cooled reactors (SCWRs) [6], and rocket engines [7]. Venugopal et al. [8] inspected the possibilities of an easy and low-cost porous insert specially designed to increase heat transmission from the heated boundary of a vertical duct under forced flow conditions. The influence of the angle of inclination and concentration of nanomaterials on mixed convection in a lid-driven cavity packed with nano-liquid was assessed by Izadi et al. [9]. The combined free-forced convection of nano-liquid through a cavity with an isolated round body was numerically investigated by Majdi et al. [10]. According to them, the local Nusselt number decreases when higher values are assigned to the Richardson number.

The study of peristaltic flow is a key research area in different fields due to its potential to provide a considerable improvement in different applications, such as biomedicine, physiology, biomedical engineering, chemical engineering, nuclear reactors, and electronics. For example, in the field of medical science, the peristalsis flow mechanism stands to execute many somatic functions, such as blood circulatory system, transportation of ovum, urine flow from the kidney to the bladder, the digestive tract, and others. Many biomedical engineering challenges, such as heart–lung machinery, and many biosystems, such as the stomach, esophagus, and digestive systems, utilize the peristaltic flow mechanism. In industrial applications, peristaltic flow is widely employed, including the transport of corrosive and sanitary fluids and the nuclear production flow of toxic liquids. The initial study of peristalsis was conducted by Latham [11] using an experimental and theoretical approach. A more detailed discussion of the peristaltic flow mechanism considering the effects of physical properties through different conduits is provided in some related works (see, for example, [12,13,14,15]).

A magnetohydrodynamic phenomenon is a physical phenomenon that describes the movement of an electrically conductive liquid in the existence of a magnetic field. Its applications have been extensively investigated across different disciplines, ranging from the study of radiosurgery applications to magnetic resonance imaging. Mekheimer et al. [16] explored the impact of space porosity and magnetic field in the presence of the peristaltic movement of Maxwell fluid via a microchannel. By considering the influences of both thermal radiation and a uniform magnetic field, Kothandapani and Prakash [17] studied the peristaltic flow past the porous tapered channel of a nanofluid. For a compressible fluid, Abumandour et al. [18] researched peristaltic flow through an elastic channel under the spell of slip conditions and magnetic intensity.

The interaction of electric fields (electroosmosis) with liquid particles and their transfer is of great interest to scientists. This analysis belongs to the category of electro-hydrodynamics (EFD). The main applications of EOF are typically found in microfluidic devices, sickle cells, processing, and chemical and soil analysis. Negative charges arise on a charged solid surface when it is mixed with an aqueous solution. Negative ions in a liquid will be repelled by these surfaces, whereas positive ions will be drawn to them. This results in the formation of an electrical double layer, which is a thin layer of imbalanced charge (EDL). The positively charged EDL will step in the direction of the electric field when it is parallel to a solid surface. Due to this, the viscous effect causes fluid to move around in the volume. Electroosmotic flow (EOF) is a highly profitable micro-pump mechanism for transferring small volumes of liquid through microchannels and capillaries. EOF has greater advantages than traditional microchannel pressure transmission. Pumps that operate on the electroosmotic mechanism can easily create high-pressure liquid flow, and these pumps are cheap, efficient in operation, and light. Rice and Whitehead [19] outlined theoretical research of electrokinetic stream in narrow cylindrical capillaries. The electroosmotic flow of a non-Newtonian fluid in a microchannel was investigated by Tang et al. [20]. The electromagnetohydrodynamic features of the peristaltic stream of a Satterby nanofluid are submitted by Ramesh and Prakash [21] through a micro-symmetric channel. A comparative study was conducted by Akram et al. [22] to study the combined flow of silver–water nanofluid and silicon dioxide–water nanofluid through a porous medium driven by electroosmosis and peristalsis. Akbar et al. [23] analyzed the heat and mass transfer for peristaltic transportation of CY nanofluid.

In both physics and technology, entropy generation (EG) plays a very essential role. It’s crucial role and use can be traced back to many areas, such as continuum physics, thermodynamics, information theory, and many others. Entropy studies as well as measures the irreversibility of systems, which is the sole cause for the allure of many investigators. In industrial thermal systems [24], EG allows us to evaluate better results and reduce energy losses. In the presence of nanofluids, the EG approach has recently been employed to analyze several industrial thermal systems [25]. The EG method is applied to provide a considerable enhancement in thermal engineering equipment. EG in natural-convection flow of nano-liquids has been explored numerically near an inverted cone by Ellahi et al. [26], and it was concluded that nanoparticle EG was formed due to nanomaterials. Sheikholislami et al. [27] researched the flow of several nanofluids within a cavity. It was found that the energy transfer can be improved by growing the volume of friction and the Rayleigh number. Khan et al. [28] expressed the EG in unsteady MHD flow via porous surfaces, incorporating the effects of heat and mass transfer. Akbar and Abbasi [29] discussed the irreversibility analysis for peristalsis of nano-liquid with variable viscosity. Zahid et al. [30] described the EG in a hybrid nano-liquid for peristalsis-driven flow. Irreversibility analysis for nano-liquid flow in an inclined channel embedded in a porous space with variable permeability was performed by Tlau and Ontela [31]. Ali et al. [32] reviewed the analysis of the second law for peristaltic transfer of variable thermal conductivity of a liquid with nonlinear convection.

The aforementioned scrupulous work ensures that heat transfer and entropy generation of an electrically conductive liquid based on kerosene due to the combined action of electroosmosis and peristalsis mechanisms have not been considered in any literature. In connection with the optimization of bio-inspired thermal energy systems, a mathematical model is being studied here to assess the generation of entropy and the efficiency of heat transfer during the current flow, together with the effects of nonlinear mixed-convective heat transfer, temperature-dependent viscosity, radiative heat flux, electric and magnetic fields, and porous medium. Basic theories are employed to develop defining expressions for a flow model. The obtained system is then resolved numerically using NDSolve in Mathematica. A detailed discussion of the respective parameters on the flow quantities is described and reflected through graphs.

## 2. Problem Formulation

### 2.1. Flow Regime

**Figure 1:**
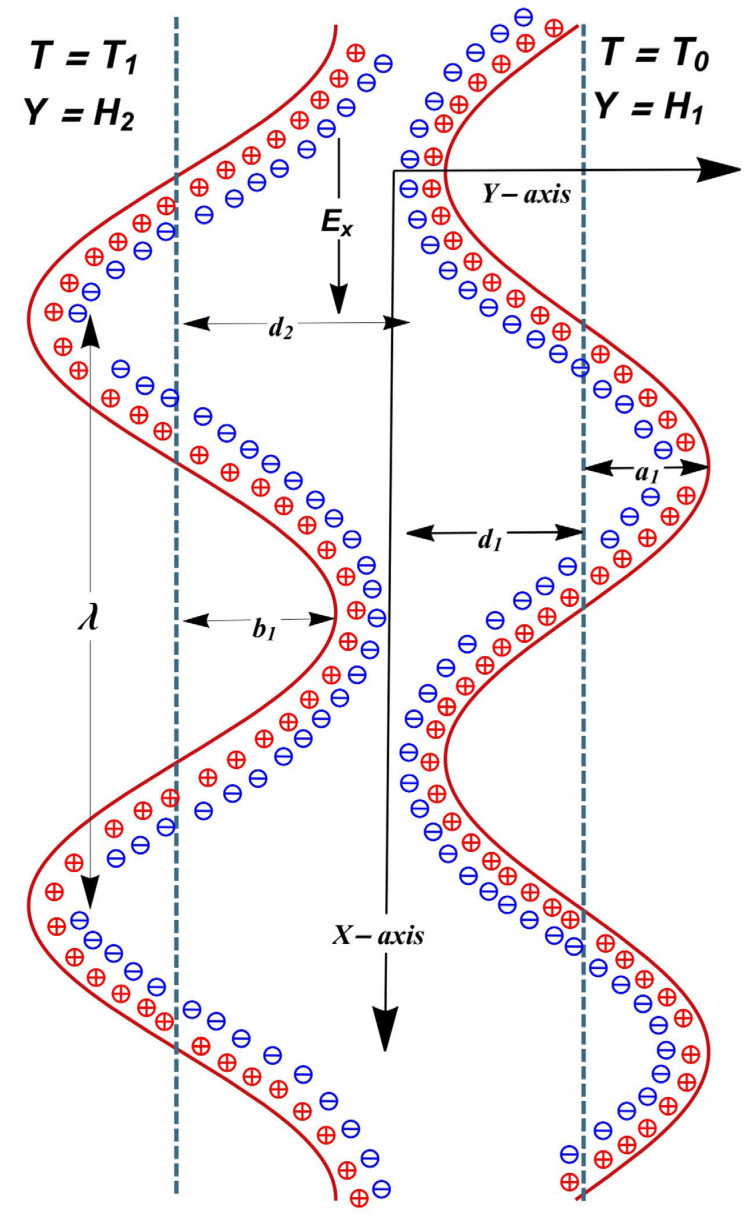
Geometry of the problem.

The electroosmotically modulated flow of magnetized liquid (kerosene) with variable viscosity inside an asymmetric channel with methodically contracting and relaxing wavy walls is mathematically interpreted. The liquid (kerosene) is considered symmetric along the z: z axis, i.e., the valence of anions and cations are the same. Electroosmotic forces are created by applying an external electric field through the EDL in the flow direction. Further, the liquid magnetization occurs due to interactions of a transverse magnetic field along the normal direction of flow. Additionally, the asymmetric channel is stuffed with porous material with variable permeability. Conveyance of liquid is inspired by the sinewave of separate phases accelerating at a uniform speed, *c*, across the channel boundaries. The wall scheme is such that $\bar{Y}={\bar{H}}_{1}\;\mathrm{and}\;{\bar{H}}_{2}$ illustrates the positions of right and left channel walls, respectively (see Figure 1). The expressions for the flow patterns on right and left boundaries of the channel are identified as:

(1) $$\begin{array}{l} \bar{H_{2}}(\bar{X},\bar{t})=-d_{2}-b_{1}\cos\left(\frac{2\pi }{\lambda }(\bar{X}-c\bar{t})+\gamma \right),\\ \bar{H_{1}}(\bar{X},\bar{t})=d_{1}+a_{1}\cos\left(\frac{2\pi }{\lambda }(\bar{X}-c\bar{t})\right), \end{array}$$

where $\lambda ,\;d_{1},\;d_{2},\;t,\;a_{1},\;\mathrm{and}\;a_{2}$ denote wavelength, channel width, time, and amplitudes, respectively. Moreover, $a_{1},\;a_{2},\;d_{1},$ $d_{2}$, and γ meet the condition:

$${\bar{d}}_{1}^{2}+{\bar{d}}_{2}^{2}+2{\bar{a}}_{1}{\bar{a}}_{2}\cos\gamma \leq {\left({\bar{d}}_{1}+{\bar{d}}_{2}\right)}^{2}.$$

For a 2D flow, the velocity, magnetic, pressure, temperature, and electrical profiles are:

$$\begin{array}{c} V=[\bar{U}(\bar{X},\bar{Y},\bar{t}),\bar{V}(\bar{X},\bar{Y},\bar{t}),0],\;\bar{P}=\bar{P}(\bar{X},\bar{Y})\\ \bar{T}=\bar{T}(\bar{X},\bar{Y},\bar{t}),\;B=\left[0,B_{0},0\right]\;\mathrm{and}\;E=\left[{\bar{E}}_{\bar{x}},0,0\right]. \end{array}$$

### 2.2. Electro and Magnetohydrodynamics

Ohmic law is listed as [30]:

(2) $$J=\sigma_{f\;}[E+V\times B].$$

The external magnetic and electric fields deemed in this problem are **E** = [*E_x_*_,_ *0*, *0*] and **B** = [*0*, *0*, *B*_0_], respectively. Lorentz force using Equation (2) becomes:

(3) $$J\times B=\left[-\bar{U}B_{0}^{2}\sigma_{f},B_{0}\sigma_{f}E_{x},0\right].$$

Due to the existence of EDL on the channel boundaries, an electric potential is created. Mathematical modeling of this layer leads to the Poisson equation [19] for the electric potential distribution, which has the form:

(4) $$\nabla^{2}\;\Omega \;¯=-\frac{\rho_{e}}{\varepsilon }.$$

The net charge density ($\rho_{e}$) obeys Boltzmann distribution [20], defined as:

(5) $$\rho_{e}=ez\left({\bar{n}}_{+}-{\bar{n}}_{-}\right),$$

where ${\bar{n}}_{+}$ is the cations and ${\bar{n}}_{-}$ is the anions. These cations and anions are specified as:

(6) $${\bar{n}}_{\pm }={\bar{n}}_{0}e^{\left(\pm \frac{ez}{T_{av}K_{B}}\;\Omega \;¯\right)}.$$

Making use of Equations (5) and (6) in Equation (4) and implementing Debye–Huckel approximation [20]:

(7) $$\frac{d^{2}\Omega }{dz^{2}}=\omega^{2}\Omega ,$$

with boundary conditions:

(8) $$\begin{array}{c} \Omega (y)=1,\;\mathrm{at}\;y=h_{2},\\ \Omega (y)=0,\;\mathrm{at}\;y=h_{1}. \end{array}$$

where ω is an electroosmotic parameter. It is described as:

(9) $$\omega =\frac{d_{1}}{\lambda_{D}},$$

where,

(10) $$\lambda_{D}=\frac{1}{ez}{\left(\frac{\varepsilon K_{B}T_{av}}{2n_{0}}\right)}^{\frac{1}{2}}.$$

The analytical solution of Equation (7) subject to boundary conditions (8) becomes:

(11) $$\Omega (y)=\frac{\sinh\left(\omega \left(y-h_{1}\right)\right)}{\sinh\left(\omega \left(h_{2}-h_{1}\right)\right)}.$$

### 2.3. Governing Equations

For the present system, the effects of nonlinear density temperature (NDT) variations, variable viscosity, variable porous medium, magnetic field, electric field, and viscous dissipation are taken into consideration. The influence of thermal radiation, including the term of the radiative heat flux, given by the Rosseland approximation, is also analyzed. Using the above supposition, the leading differential equations in a laboratory structure are described as [29,30,31,32]:

(12) $$\frac{\partial \bar{U}}{\partial \bar{X}}+\frac{\partial \bar{V}}{\partial \bar{Y}}=0,$$

(13) $$\begin{array}{r} \rho_{f}\left(\bar{U}\frac{\partial }{\partial \bar{X}}+\frac{\partial }{\partial \bar{t}}+\bar{V}\frac{\partial }{\partial \bar{Y}}\right)\bar{U}=-\frac{\partial \bar{P}}{\partial \bar{X}}+\frac{\partial }{\partial \bar{Y}}\left(\bar{\mu }(T)\right)\left[\frac{\partial \bar{U}}{\partial \bar{Y}}+\frac{\partial \bar{V}}{\partial \bar{X}}\right]+2\frac{\partial }{\partial \bar{X}}\left[\bar{\mu }(T)\frac{\partial \bar{U}}{\partial \bar{X}}\right]+\rho_{e}{\bar{E}}_{x}\\ -\bar{U}B_{0}{}^{2}\sigma_{f}-\frac{\bar{\mu }(T)}{\tilde{k}\left(\bar{Y}\right)}\bar{U}+\rho g\left[\beta_{0}\left(T-T_{m}\right)+\beta_{1}{\left(T-T_{m}\right)}^{2}\right], \end{array}$$

(14) $$\begin{array}{r} \rho_{f}\left(\frac{\partial \bar{V}}{\partial \bar{t}}+\bar{U}\frac{\partial \bar{V}}{\partial \bar{X}}+\bar{V}\frac{\partial \bar{V}}{\partial \bar{Y}}\right)=-\frac{\partial \bar{P}}{\partial \bar{Y}}+\frac{\partial }{\partial \bar{X}}\left(\bar{\mu }(T)\right)\left[\frac{\partial \bar{V}}{\partial \bar{Y}}+\frac{\partial \bar{U}}{\partial \bar{X}}\right]+2\frac{\partial }{\partial \bar{Y}}\left[\bar{\mu }(T)\frac{\partial \bar{U}}{\partial \bar{X}}\right]\\ -\frac{\bar{\mu }(T)}{\tilde{k}\left(\bar{Y}\right)}\bar{V}+{\bar{E}}_{x}B_{0}\sigma_{f}, \end{array}$$

(15) $$\begin{array}{r} {\left(\rho C\right)}_{f}\left(\bar{U}\frac{\partial }{\partial \bar{X}}+\frac{\partial }{\partial \bar{t}}+\bar{V}\frac{\partial }{\partial \bar{Y}}\right)T=K_{f}\left(\frac{\partial^{2}T}{\partial {\bar{X}}^{2}}+\frac{\partial^{2}T}{\partial {\bar{Y}}^{2}}\right)+\Phi -\frac{\partial q_{r}}{\partial \bar{Y}}+\sigma_{f}{\left(E_{x}\right)}^{2}+\frac{\bar{\mu }(T)}{\tilde{k_{0}}\left(\bar{Y}\right)}\left({\bar{U}}^{2}+{\bar{V}}^{2}\right)\\ +\sigma_{f}{\left(\bar{U}B_{0}\right)}^{2}+\bar{\mu }(T)\left({\left(\frac{\partial \bar{V}}{\partial \bar{X}}+\frac{\partial \bar{U}}{\partial \bar{Y}}\right)}^{2}+2{\left(\frac{\partial \bar{U}}{\partial \bar{X}}\right)}^{2}+2{\left(\frac{\partial \bar{V}}{\partial \bar{Y}}\right)}^{2}\right), \end{array}$$

In the above equations, $T,\;\bar{P}$, $\rho_{f}$, $T_{m}$, and Φ are, respectively, the fluid’s temperature, pressure, density, mean temperature, and heat absorption/generation.

**Figure 2:**
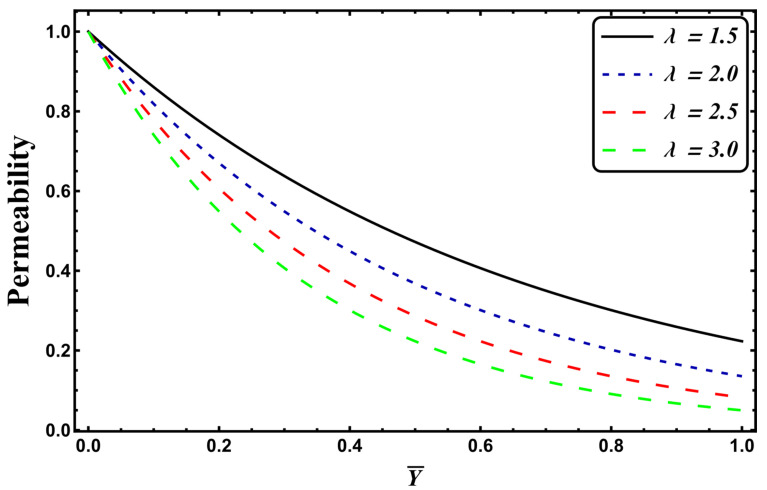
Variation in permeability.

It is assumed that the permeability of the porous medium changes exponentially over the channel width, since the flow is considered to be laminar. With such assumptions, a variable permeability of the porous medium is stated as [31]:

(16) $$\tilde{k}\left(\bar{Y}\right)={\tilde{k}}_{0}e^{-\lambda }\bar{Y}$$

where $\tilde{k_{0}}$ is the permeability (constant) and λ is the permeability parameter. Figure 2 shows that an upsurge in the permeability parameter leads to reduced permeability of the porous media.

To convert the governing Equations (12)–(15) from a laboratory frame ($\bar{X},\bar{Y},\bar{t})$ to a wave frame ($\bar{x},\bar{y})$, the following transformations are used [32]:

(17) $$\bar{v}=\bar{V},\;\bar{x}=\bar{X}-c\bar{t},\;\bar{p}\left(\bar{x},\bar{y}\right)=\bar{P}\left(\bar{X},\bar{Y},\bar{t}\right),\;\bar{y}=\bar{Y},\;\bar{u}=\bar{U}-c.$$

In the above equation, $\bar{u}$ and $\bar{v}$ are, respectively, the $\bar{x}$ and $\bar{y}$ components of velocity in the wave frame. In the wave frame, Equations (12)–(15) take the following form:

(18) $$\frac{\partial \bar{u}}{\partial \bar{x}}+\frac{\partial \bar{v}}{\partial \bar{y}}=0,$$

(19) $$\frac{\partial \bar{u}}{\partial \bar{x}}+\frac{\partial \bar{v}}{\partial \bar{y}}=0,\begin{array}{r} \rho_{f}\left(\bar{v}\frac{\partial }{\partial \bar{y}}+(\bar{u}+c)\frac{\partial }{\partial \bar{x}}\right)(\bar{u}+c)=-\frac{\partial \bar{P}}{\partial \bar{x}}+\frac{\partial }{\partial \bar{y}}\left(\bar{\mu }(T)\right)\left[\frac{\partial \bar{u}}{\partial \bar{y}}+\frac{\partial \bar{v}}{\partial \bar{x}}\right]+2\frac{\partial }{\partial \bar{x}}\left[\bar{\mu }(T)\frac{\partial \bar{u}}{\partial \bar{x}}\right]+\rho_{e}{\bar{E}}_{x}\\ -(\bar{u}+c)B_{0}{}^{2}\sigma_{f}-\frac{\bar{\mu }(T)}{\tilde{k}\left(\bar{y}\right)}(\bar{u}+c)+\rho g\left[\beta_{0}\left(T-T_{m}\right)+\beta_{1}{\left(T-T_{m}\right)}^{2}\right], \end{array}$$

(20) $$\rho_{f}\left(\bar{v}\frac{\partial }{\partial \bar{y}}+(c+\bar{u})\frac{\partial }{\partial \bar{x}}\right)\bar{v}=-\frac{\partial \bar{p}}{\partial \bar{y}}+\frac{\partial }{\partial \bar{x}}\left(\bar{\mu }(T)\right)\left[\frac{\partial \bar{v}}{\partial \bar{y}}+\frac{\partial \bar{u}}{\partial \bar{x}}\right]+2\frac{\partial }{\partial \bar{y}}\left[\bar{\mu }(T)\frac{\partial \bar{u}}{\partial \bar{x}}\right]-\frac{\bar{\mu }(T)}{\tilde{k}\left(\bar{y}\right)}\bar{v}+{\bar{E}}_{x}B_{0}\sigma_{f},$$

(21) $$\begin{array}{r} {\left(\rho C\right)}_{f}\left(\bar{v}\frac{\partial }{\partial \bar{y}}+\left(c+\bar{u}\right)\frac{\partial }{\partial \bar{x}}\right)T=K_{f}\left(\frac{\partial^{2}T}{\partial {\bar{x}}^{2}}+\frac{\partial^{2}T}{\partial {\bar{y}}^{2}}\right)+\Phi -\frac{\partial q_{r}}{\partial \bar{y}}+\sigma_{f}{\left(E_{x}\right)}^{2}+\frac{\bar{\mu }(T)}{\tilde{k_{0}}\left(\bar{y}\right)}\left({\left(c+\bar{u}\right)}^{2}+{\bar{v}}^{2}\right)\\ +\frac{16}{3}\frac{\sigma^{*}}{k^{*}}T_{0}^{3}\frac{\partial^{2}T}{\partial {\bar{y}}^{2}}+\sigma_{f}{\left(\left(c+\bar{u}\right)B_{0}\right)}^{2}+\bar{\mu }(T)\left({\left(\frac{\partial \bar{v}}{\partial \bar{x}}+\frac{\partial \bar{u}}{\partial \bar{y}}\right)}^{2}+2{\left(\frac{\partial \bar{u}}{\partial \bar{x}}\right)}^{2}+2{\left(\frac{\partial \bar{v}}{\partial \bar{y}}\right)}^{2}\right), \end{array}$$

The reliance of the viscosity of the liquid on the temperature is offered as [29]:

(22) $$\bar{\mu }(T)=\mu_{0}\left(1+\alpha_{0}\left(T-T_{m}\right)\right).$$

### 2.4. Dimensionless Quantities

The dimensionless quantities are defined as follows:

(23) x=x¯λ, t=ct¯λ, h1=H1d1, h2=H2d1, a=a1d1, b=b1d1, d=d2d1,  p=d12p¯cλμ0, υ=μ0ρf, y=y¯d1, v=−ψx,v=v¯cδ, δ=d1λ, α=α0(T1−T0), M2=σfμ0B02d12, u=u¯c, Re=ρfcd1μ0,Pr=μ0CfKf, Br=Pr.Ec, Ec=c2Cf(T1−T0), θ=T−TmT1−T0, ε=Φd12(T1−T0)Kf, u=ψy, S=d12σfEx2Kf(T1−T0), Da=k˜0d12, R=d1λ,λ1=β1(T1−T0)β0, Gr=ρgd12β0(T−T0)μ0c, Uhs=−εExμ0c, Nr=163σ∗k∗T03Kf. 

Invoking the above-mentioned dimensionless quantities (23) and applying “small Reynolds number and large wavelength approximations” in Equations (18)–(21), we have:

(24) $$\begin{array}{r} 0=-\frac{\partial p}{\partial x}+\frac{\partial }{\partial y}\left(\left(1-\alpha \theta \right)\frac{\partial^{2}\psi }{\partial y^{2}}\right)+Gr\theta \left(1+\lambda_{1}\theta \right)-\frac{1}{e^{-Ry}}\left[\frac{\left(1-\alpha \theta \right)}{Da}\left(1+\frac{\partial \psi }{\partial y}\right)\right]\\ -M^{2}\left(\frac{\partial \psi }{\partial y}+1\right)+U_{hs}\Omega^{\prime \prime }(y), \end{array}$$

(25) $$\frac{\partial p}{\partial y}=0.$$

Disregarding the pressure gradient from Equations (24) and (25) by cross-differentiating, the resulting equation yields:

(26) $$\begin{array}{r} 0=\frac{\partial^{2}}{\partial y^{2}}\left(\left(1-\alpha \theta \right)\frac{\partial^{2}\psi }{\partial y^{2}}\right)+Gr\left[\frac{\partial \theta }{\partial y}\left(1+\lambda_{1}\theta \right)+\lambda_{1}\theta \frac{\partial \theta }{\partial y}\right]-M^{2}\frac{\partial^{2}\psi }{\partial y^{2}}+U_{hs}\Omega^{\prime \prime \prime }(y)\\ -\frac{\partial }{\partial y}\left[\frac{1}{e^{-Ry}}\left(\frac{\left(1-\alpha \theta \right)}{Da}\left(1+\frac{\partial \psi }{\partial y}\right)\right)\right], \end{array}$$

The dimensionless energy, concentration equations, and stress component take the following form:

(27) $$\frac{\partial^{2}\theta }{\partial y^{2}}+Br\left(1-\alpha \theta \right){\left(\frac{\partial^{2}\psi }{\partial y^{2}}\right)}^{2}+BrM^{2}{\left(1+\frac{\partial \psi }{\partial y}\right)}^{2}+S+\frac{Br}{e^{-Ry}}\frac{\left(1-\alpha \theta \right)}{Da}{\left(1+\frac{\partial \psi }{\partial y}\right)}^{2}+Nr\frac{\partial^{2}\theta }{\partial y^{2}}+\varepsilon =0.$$

In these equations, *Pr* is the Prandtl number, *Da* is the Darcy number, $W_{e}$ is the Weissenberg number, *Br* is the Brinkman number, *M* is the Hartman parameter, and ε is the heat source/sink parameter.

The connection between dimensionless volume flow rates in fixed η and moving F frames is [29]:

(28) η=F+1+d,

here, *F* is given as:

$$F=\int_{h_{2}}^{h_{1}}\frac{\partial \psi }{\partial y}dy.$$

### 2.5. Boundary Conditions

The dimensionless boundary conditions in the wave frame are listed as:

(29) $$\psi =\frac{F}{2},\;\frac{\partial \psi }{\partial y}=-1,\;\theta =-\frac{1}{2}\;\mathrm{at}\;y=h_{1},\;\psi =-\frac{F}{2},\;\frac{\partial \psi }{\partial y}=-1,\;\theta =\frac{1}{2}\;\mathrm{at}\;y=h_{2}.$$

## 3. Entropy Generation Analysis

The dimensional form of the volumetric entropy generation rate is illustrated as:

(30) $$\begin{array}{l} S_{G}=\underset{\mathrm{Thermal}\;\mathrm{irreversibility}}{\underset{︸}{\frac{K_{f}}{{\left(T_{1}-T_{0}\right)}^{2}}\left[{\left(\frac{\partial T}{\partial \bar{X}}\right)}^{2}+{\left(\frac{\partial T}{\partial \bar{Y}}\right)}^{2}\right]}}++\underset{\mathrm{Fluid}\;\mathrm{Friction}\;\mathrm{irreversibility}}{\underset{︸}{\frac{\bar{\mu }(T)}{T_{1}-T_{0}}\left(\begin{array}{l} {\left(\frac{\partial \bar{V}}{\partial \bar{X}}+\frac{\partial \bar{U}}{\partial \bar{Y}}\right)}^{2}\\ 2\left\{{\left(\frac{\partial \bar{V}}{\partial \bar{Y}}\right)}^{2}+{\left(\frac{\partial \bar{U}}{\partial \bar{X}}\right)}^{2}\right\} \end{array}\right)}}+\underset{\mathrm{Electric}\;\mathrm{field}\;\mathrm{irreversibility}}{\underset{︸}{\frac{1}{T_{1}-T_{0}}\sigma_{f}{\left(E_{x}\right)}^{2}}}\\ \underset{\begin{array}{l} \mathrm{Heat}\;\mathrm{generation}/\mathrm{absorption}\\ \;\mathrm{irreversibility} \end{array}}{\underset{︸}{\frac{\Phi }{T_{1}-T_{0}}}}+\underset{\mathrm{Magnetic}\;\mathrm{field}\;\mathrm{irreversibility}}{\underset{︸}{\frac{1}{T_{1}-T_{0}}\sigma_{f}{\left(\bar{U}B_{0}\right)}^{2}}}+\underset{\mathrm{Thermal}\;\mathrm{radiation}\;\mathrm{irreversibility}\;}{\underset{︸}{\frac{1}{T_{1}-T_{0}}\frac{16}{3}\frac{\sigma^{*}}{k^{*}}T_{0}^{3}{\left(\frac{\partial T}{\partial \bar{Y}}\right)}^{2}}}+\underset{\mathrm{Porous}\;\mathrm{medium}\;\mathrm{irreversibility}}{\underset{︸}{\frac{1}{T_{1}-T_{0}}\frac{\bar{\mu }(T)}{\tilde{k_{1}}\left(\bar{Y}\right)}\left({\bar{U}}^{2}+{\bar{V}}^{2}\right)}}, \end{array}$$

Equation (30) consists of seven physical quantities that cause the generation of entropy, i.e., impacts of heat transfer due to radiation and conduction, the effects of viscous dissipation, heat generation/absorption, electric field, porous medium, and magnetic field. The dimensionless entropy generation number, $N_{s}$, is written as:

(31) $$N_{S}=\frac{S_{G}}{S_{c}},\;\begin{array}{r} N_{S}=\left(1+Nr\right){\left(\frac{\partial \theta }{\partial y}\right)}^{2}+Br\left(1-\alpha \theta \right){\left(\frac{\partial^{2}\psi }{\partial y^{2}}\right)}^{2}+BrM^{2}{\left(1+\frac{\partial \psi }{\partial y}\right)}^{2}+S+\varepsilon \\ +\frac{Br}{e^{-Ry}}\frac{\left(1-\alpha \theta \right)}{Da}{\left(1+\frac{\partial \psi }{\partial y}\right)}^{2}, \end{array}$$

where $S_{c}$ is the characteristic entropy generation rate, and is identified as:

$$S_{c}=\frac{K_{f}}{d_{1}{}^{2}}.$$

Moreover, the Bejan number, $B_{e}$, is defined as “the ratio of entropy generation due to heat transfer to the total entropy generation”. Mathematically, it is written as:

(32) $$B_{e}=\frac{\left(1+Nr\right){\left(\frac{\partial \theta }{\partial y}\right)}^{2}}{\left(1+Nr\right){\left(\frac{\partial \theta }{\partial y}\right)}^{2}+Br\left(1-\alpha \theta \right){\left(\frac{\partial^{2}\psi }{\partial y^{2}}\right)}^{2}+BrM^{2}{\left(1+\frac{\partial \psi }{\partial y}\right)}^{2}+S+\varepsilon +\frac{Br}{e^{-Ry}}\frac{\left(1-\alpha \theta \right)}{Da}{\left(1+\frac{\partial \psi }{\partial y}\right)}^{2}}.$$

The Bejan number varies from 0 to 1.

## 4. Results and Discussion

The present section is dedicated to explaining the results obtained through graphs and tables. To determine thermal efficiency and entropy production, we examined the effects of an extensive range of parameters on fluid temperature, heat transfer rate at the channel center and boundary, entropy generation, Bejan number, and fluid velocity. Throughout our numerical computation, the default values of various non-dimensional pertinent parameters are assigned as: a=0.6, Gr=2, $\gamma =\frac{\pi }{4},$ b=0.7, m=1, d=0.8, η=0.9, x=1, M=2, ϵ=1, R=1.2, Da=0.5, Nr=1, ω=2, $U_{hs}=-1,$ S=0.5, M=1, α=0.03, α=0.03, Pr=21, $\lambda_{1}=0.1$, and Br=0.3, unless stated otherwise.

### 4.1. Temperature Profile

**Figure 3:**
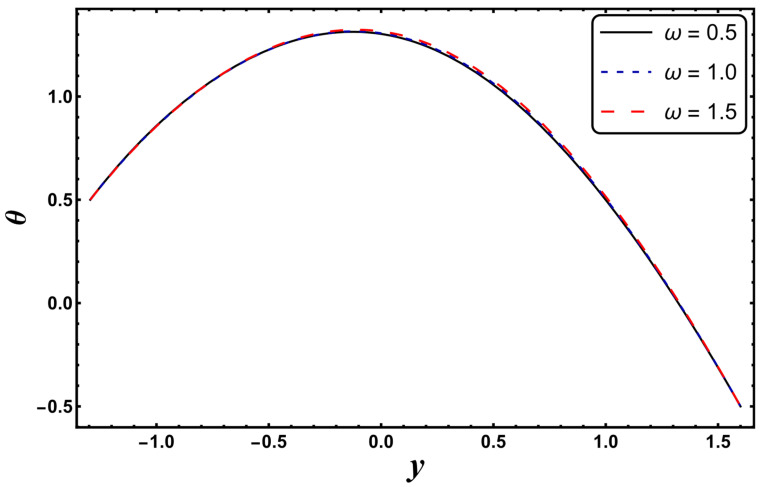
*θ* for variation in *ω*.

**Figure 4:**
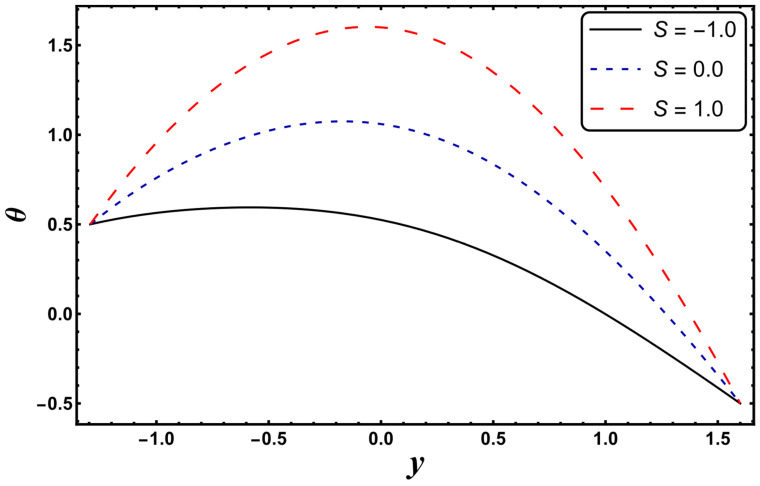
*θ* for variation in *S*.

**Figure 5:**
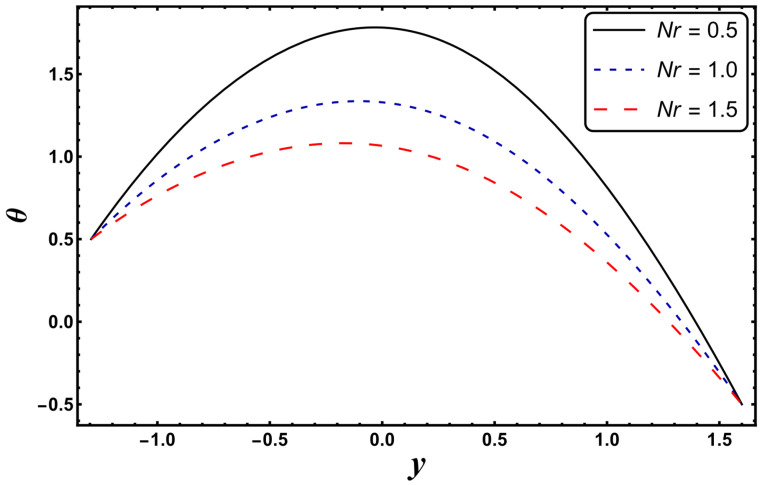
*θ* for variation in *Nr*.

**Figure 6:**
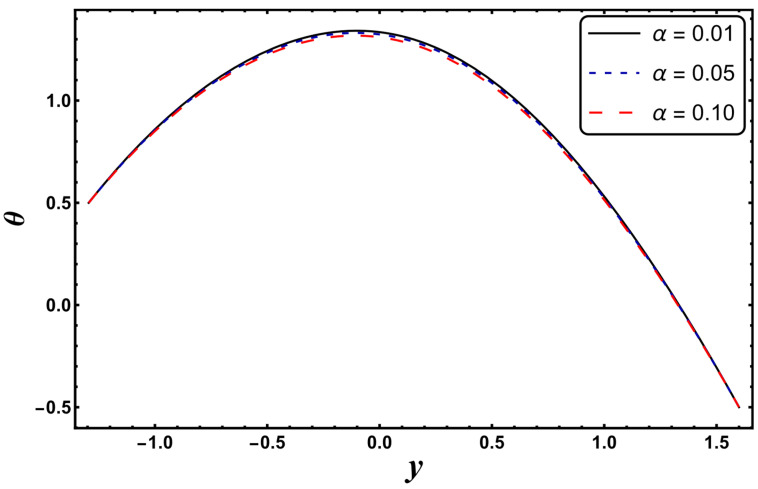
*θ* for variation in *α*.

**Figure 7:**
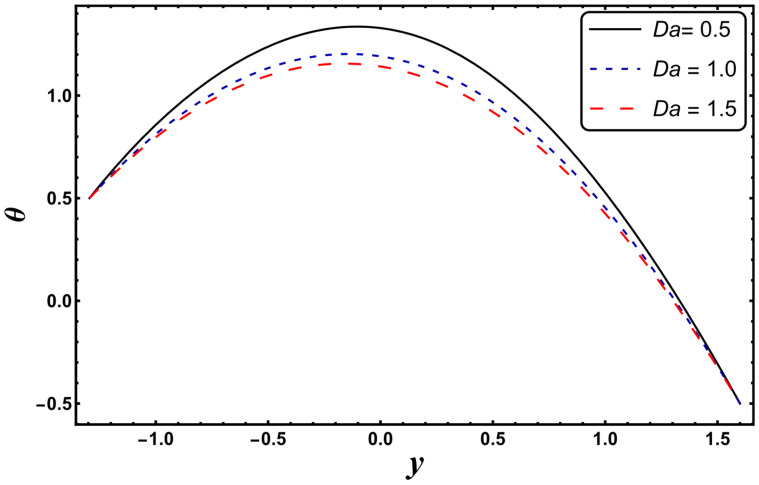
*θ* for variation in *Da*.

**Figure 8:**
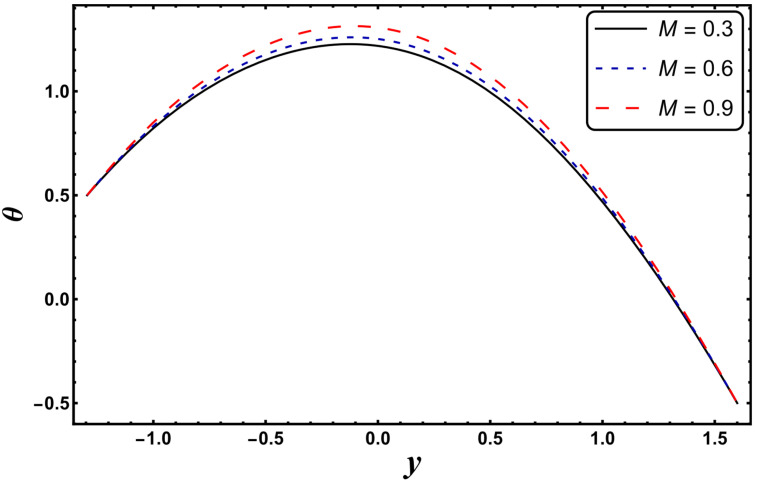
*θ* for variation in *M*.

**Figure 9:**
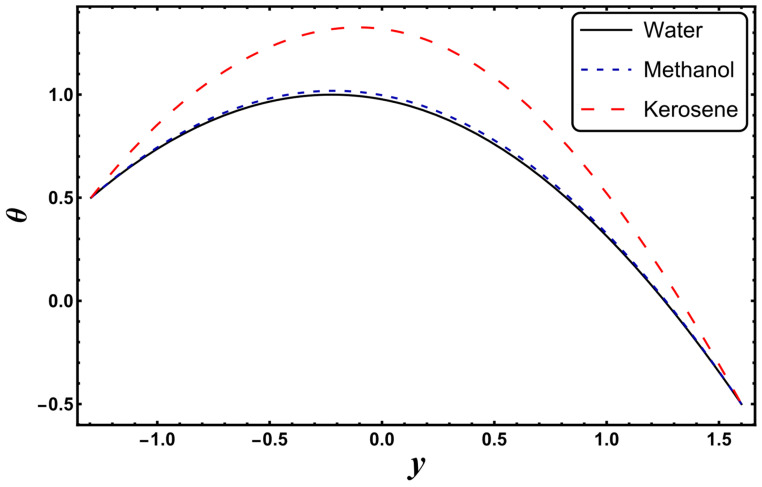
*θ* for different base fluids.

Figure 3, Figure 4, Figure 5, Figure 6, Figure 7 and Figure 8 exemplify the repercussions of several involved pertinent parameters on the temperature profile. These figures show that the magnitude of temperature is maximum at the center of the channel, whereas it attains a minimum value near the vicinity of the channel. Figure 3 exhibits that the temperature elevates with an increment in the electroosmotic parameter, *ω*. It is acknowledged that the electroosmotic force is the force of resistance to the flow, it enhances the collision among particles of the liquid. An increase in collision increases the internal kinetic energy of the carrying particles in the direction of flow, and hence, an increase in temperature is reflected. Figure 4 supports a considerable upsurge in temperature by augmenting *S*. Physically, this is owing to the dissipated electrical energy being converted into heat. In contrast, Figure 5 signifies that temperature is a decaying function of *Nr*. A similar result was also observed by Kothandapani and Prakash [17]. Figure 5 elucidates that temperature suppresses for a higher *α*. The physical reason for this activity is that an intensification in *α* enhances the heat transfer capacity, which facilitates the rapid removal of heat from the system. It is shown from Figure 7 that temperature decreases upon increasing the Darcy number, *Da*. This behavior is physically valid because when a fluid flows through a porous medium, internal energy is dispelled by friction between the fluid and the porous material, that eventually decreases the temperature. Figure 8 reveals that amplification of the Hartmann number, *M*, augments the temperature distribution. The fluid has to expend more work to drag itself against the effect of the retarding magnetic field. This additional exertion is dissipated as thermal energy, which heats up the system. This result is consistent with Abdelsalam and Bhatti’s work [13]. Figure 9 was designed to evaluate and compare the temperature for different base fluids. It was observed that temperature is comparatively lower for water compared to kerosene and methanol.

### 4.2. Heat Transfer Rate at the Wall

**Figure 10:**
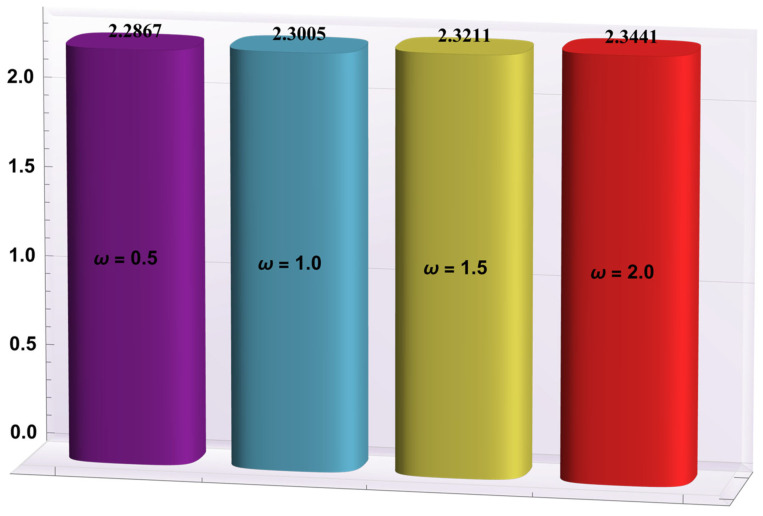
$-\theta^{\prime }\left(h_{1}\right)$ for variation in *ω*.

**Figure 11:**
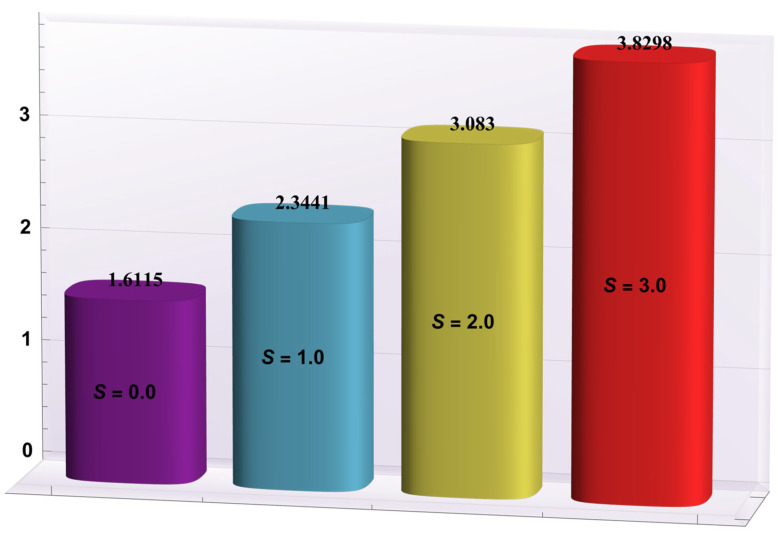
$-\theta^{\prime }\left(h_{1}\right)$ for variation in *S*.

**Figure 12:**
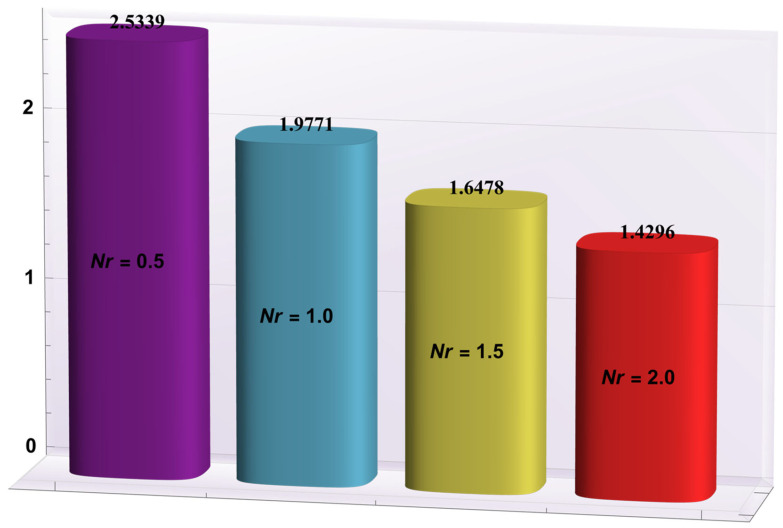
$-\theta^{\prime }\left(h_{1}\right)$ for variation in *Nr*.

**Figure 13:**
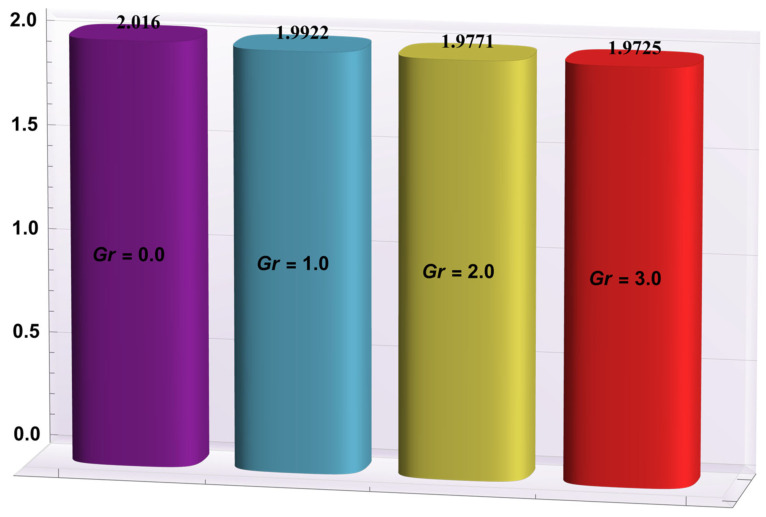
$-\theta^{\prime }\left(h_{1}\right)$ for variation in *Gr*.

**Figure 14:**
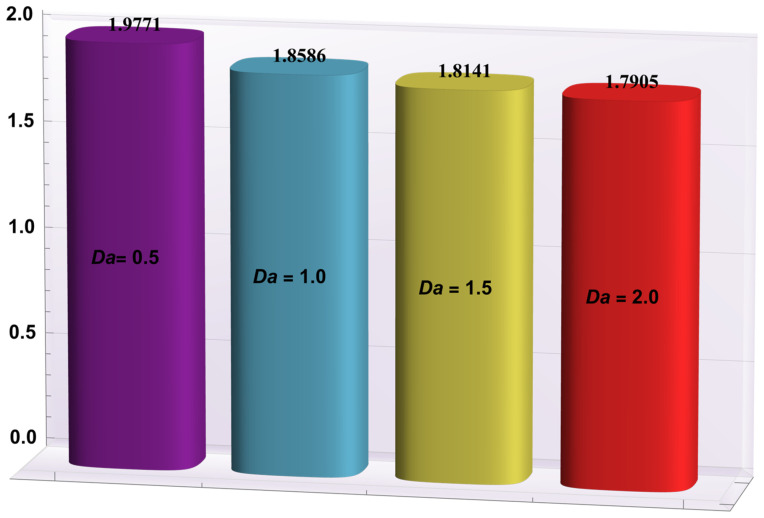
$-\theta^{\prime }\left(h_{1}\right)$ for variation in *Da*.

**Figure 15:**
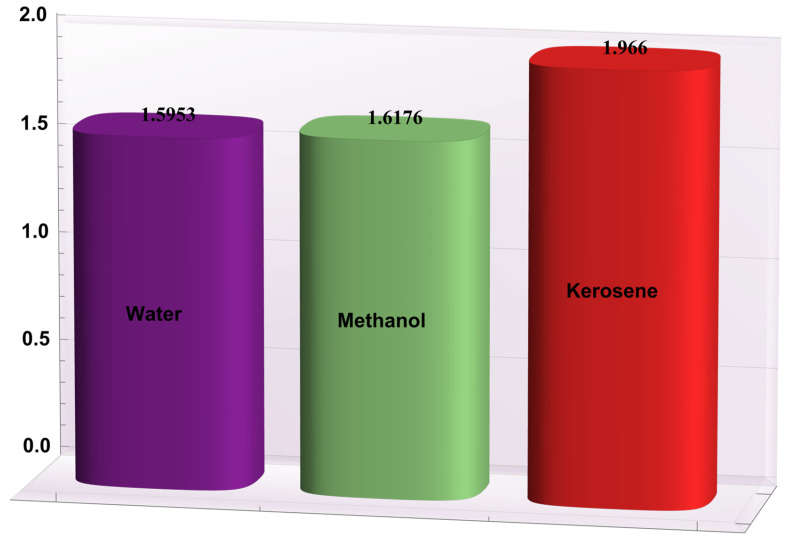
$-\theta^{\prime }\left(h_{1}\right)$ for different base fluids.

Here, the deviations of some of the arising parameters on the rate of heat transfer at the right wall, $-\theta^{\prime }\left(h_{1}\right),$ are investigated. For this purpose, bar charts were designed and revealed through Figure 10, Figure 11, Figure 12, Figure 13 and Figure 14. Figure 10 shows that electroosmotic phenomena enhance the $-\theta^{\prime }\left(h_{1}\right)$ when set in such a manner that peristaltic pumping is facilitated. An advancement in $-\theta^{\prime }\left(h_{1}\right)$ was also encountered for a higher *S* (see Figure 11). This result shows that *S*, with its increasing values, enhances the phenomenon of heat transmission between the solid boundary and the base liquid. From Figure 12, a contrary behavior is witnessed for greater values of *Nr*. This is mainly due to radiation effects that prevent the fluid temperature from rising. Consequently, there was a decrease in $-\theta^{\prime }\left(h_{1}\right)$. From Figure 13, it is seen that $-\theta^{\prime }\left(h_{1}\right)$ is a decreasing function of *Gr*. A similar tendency was also seen by Akbar and Abbasi [20]. Figure 14 delineates that *Da*, with its increasing values, lowers the $-\theta^{\prime }\left(h_{1}\right)$, which can be useful in electronic cooling applications. Figure 15 was made to compare the $-\theta^{\prime }\left(h_{1}\right)$ for three distinct base liquids (water, methanol, and kerosene). It was determined that kerosene has a higher $-\theta^{\prime }\left(h_{1}\right)$ compared to water and methanol.

### 4.3. Temperature at Channel Center

Numerical values of the maximum liquid temperature for changing several involved parameters are shown in Table 1. This table also compares the maximum temperatures of water, methanol, and kerosene. It can be seen from this table that kerosene has a higher temperature as compared to other base fluids. Further, a considerable decrease in temperature at the channel center is noted for a higher *α*, *Nr*, and *Da*, and it increases for a larger *Gr* and *S*.

### 4.4. Entropy Generation

**Figure 16:**
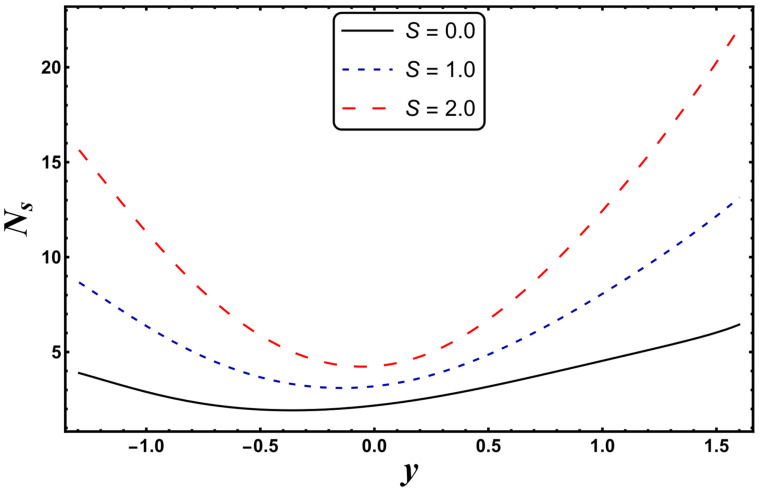
*N_s_* for variation in *S*.

**Figure 17:**
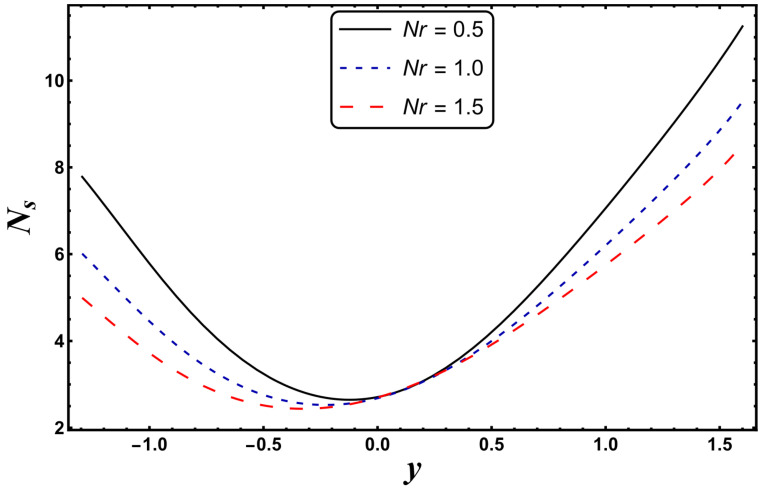
*N_s_* for variation in *Nr*.

**Figure 18:**
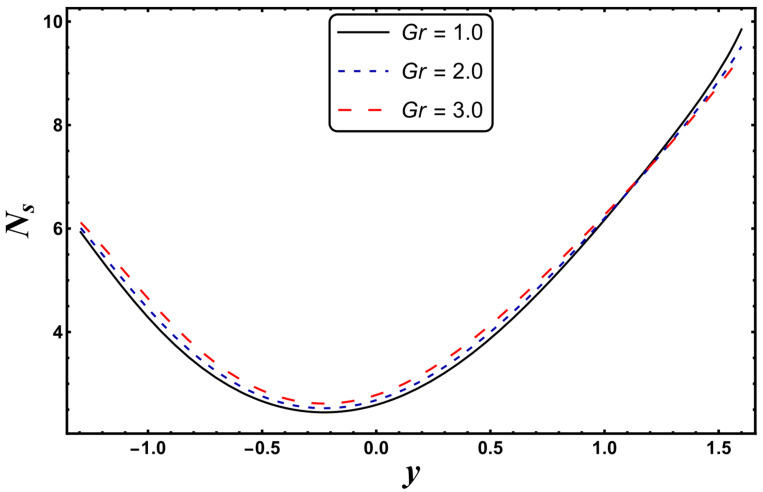
*N_s_* for variation in *Gr*.

**Figure 19:**
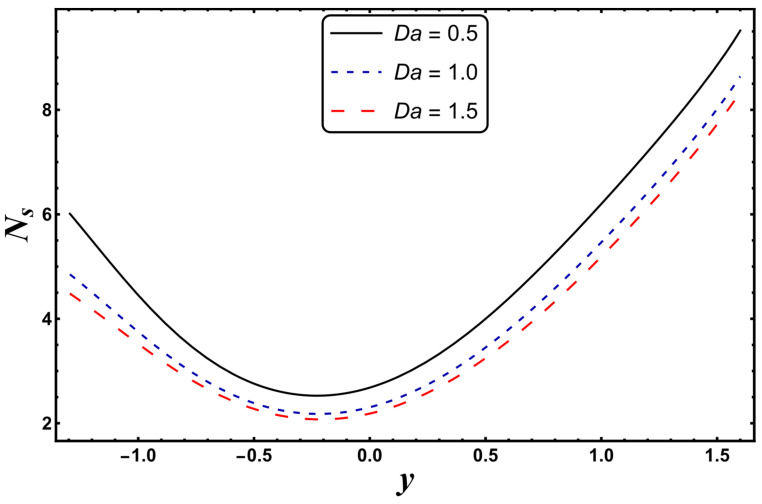
*N_s_* for variation in *Da*.

**Figure 20:**
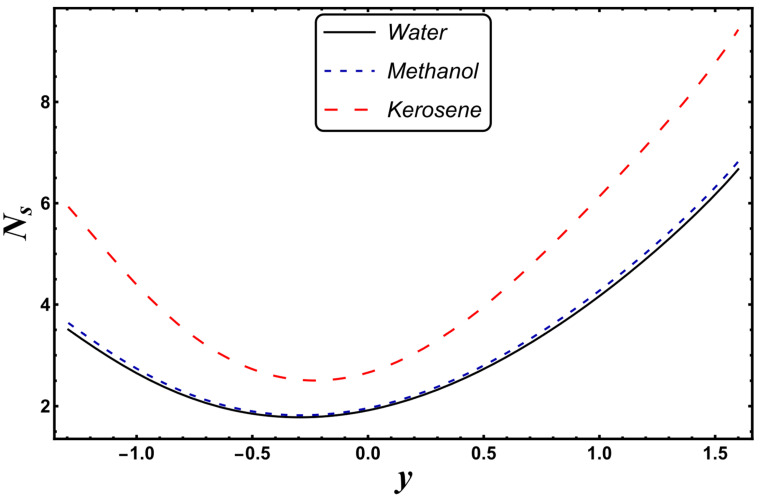
*N_s_* for different base fluids.

The most engrossing part of this section is the analysis of entropy generation (irreversibility). Figure 16, Figure 17, Figure 18 and Figure 19 elaborate on the analysis of entropy or randomness in a system for the variation in different involved parameters. An inspection of all these graphs clearly reveals that the production of entropy was very small in the middle of the parabolic curve and extensively high at its sides. It is inferred from Figure 16 that the entropy generation grows quickly due to a higher Joule heating parameter, *S*. Since *S* is proportional to the square of the electric field, therefore, an intense electric field leads to an augmentation in thermal energy. Since entropy is directly associated with temperature, therefore, entropy enhances with increasing temperature. Figure 16 also demonstrates that the impact of *S* is very strong near the boundaries of the channel. Figure 17 designates that entropy undergoes a slight reduction for a higher *Nr*. Since *Nr* is inversely proportional to the heat absorption coefficient, k*, thus, the absorption parameter declines for more radiation. It is obvious that extra heat is radiated, and the entropy is lessened. This concludes that the radiation parameter plays a significant role in the entropy generation minimization process. Figure 18 suggests that a growth in *N_s_* is seen with increasing *Gr*. However, in the neighborhood of the right wall, this behavior is the opposite. This can be illustrated as the mixed convection helps the fluid reach a higher temperature, which corresponds to a higher entropy (as also reported by Qasim et al. [32]). On the other hand, *Da*, with its higher values, serves to reduce the generation of entropy (see Figure 19). Figure 20 shows the behavior of entropy for different base fluids. The acquired result exposes that kerosene has a higher entropy, while water has a minimal entropy. Such a finding is extremely beneficial in the mechanism of mechanical equipment.

### 4.5. Bejan Number

**Figure 21:**
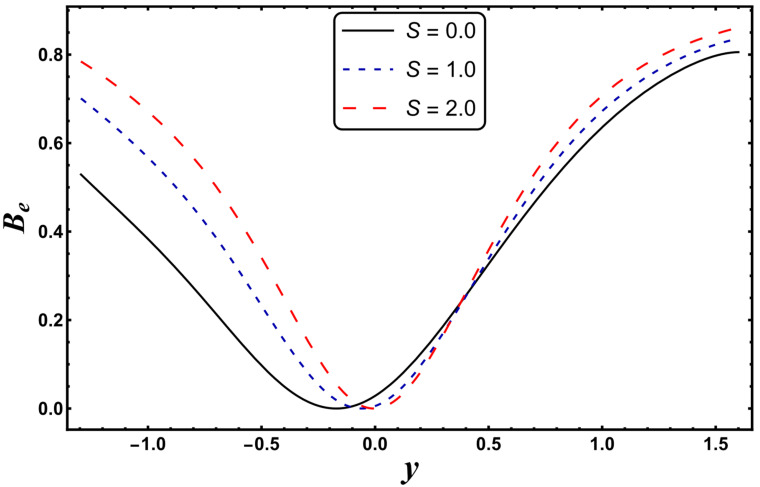
*B_e_* for variation in *S*.

**Figure 22:**
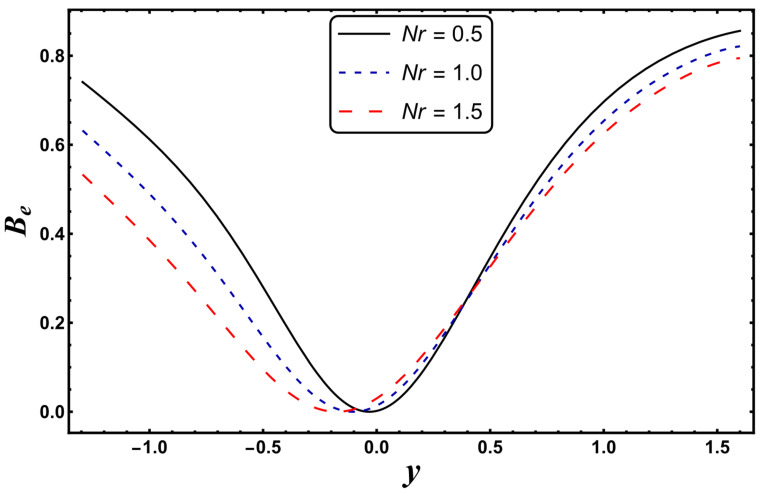
*B_e_* for variation in *Nr*.

**Figure 23:**
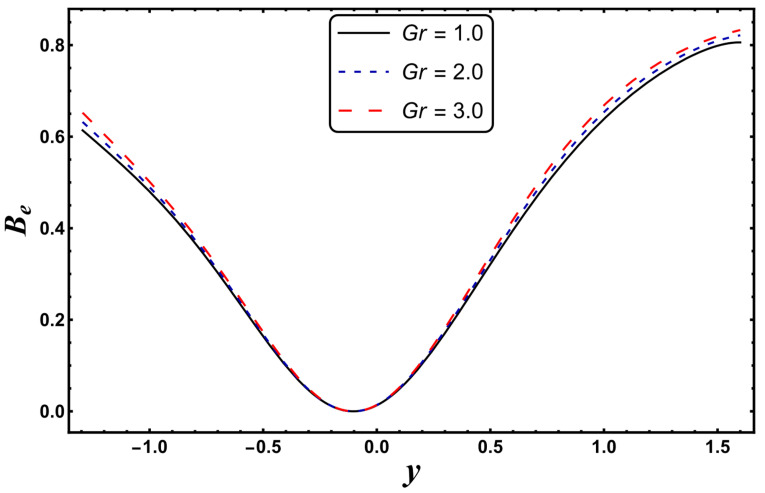
*B_e_* for variation in *Gr*.

**Figure 24:**
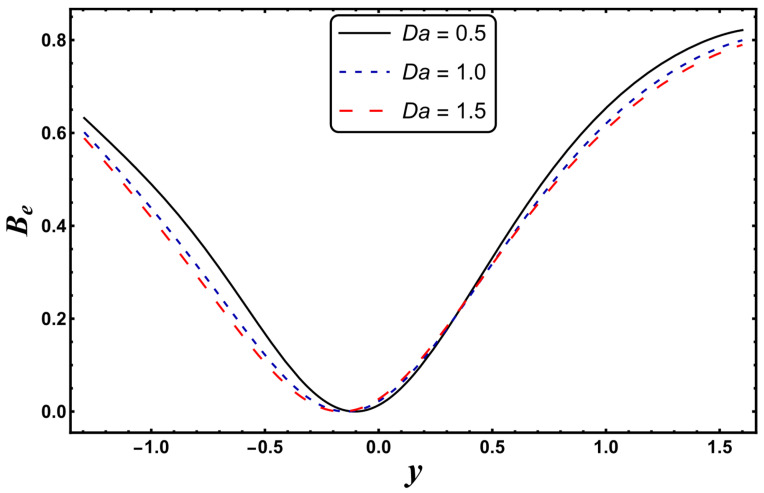
*B_e_* for variation in *Da*.

Figure 21, Figure 22, Figure 23 and Figure 24 are schematically shown to clarify the influence of various emerging physical parameters, i.e., *S*, *Nr*, *Gr*, and *Da*, on the Bejan number. The Bejan number rises in the vicinity of channel walls for a higher *S* (see Figure 21). This is mainly due to the dominance of thermal irreversibility compared to total irreversibility, which favors the augmentation of the Bejan number. It is seen from Figure 22 that the Bejan number falls for the case of a higher *Nr*. A similar tendency is rendered for the case of a higher *Da* (see Figure 23). Here, a decrease in the Bejan number means that the total irreversibility exceeds the irreversibility caused by thermal conduction. The Bejan number increases for a higher *Gr* (see Figure 24).

### 4.6. Velocity Profile

**Figure 25:**
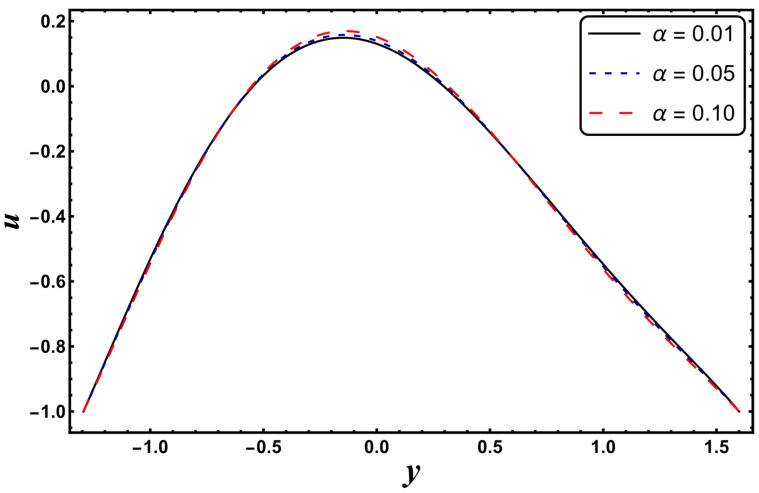
*u* for variation in *α*.

**Figure 26:**
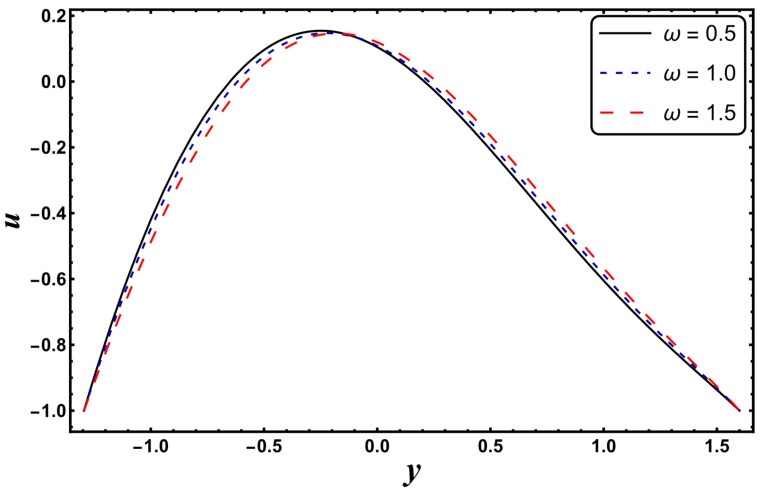
*u* for variation in *ω*.

**Figure 27:**
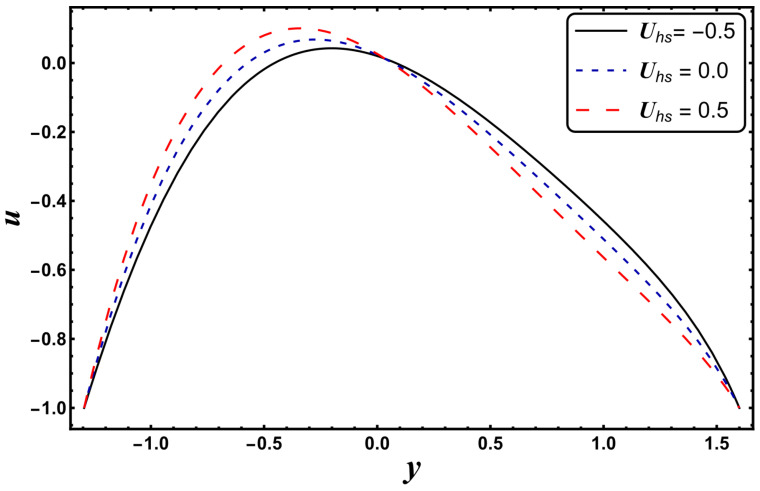
*u* for variation in *U_hs_*.

**Figure 28:**
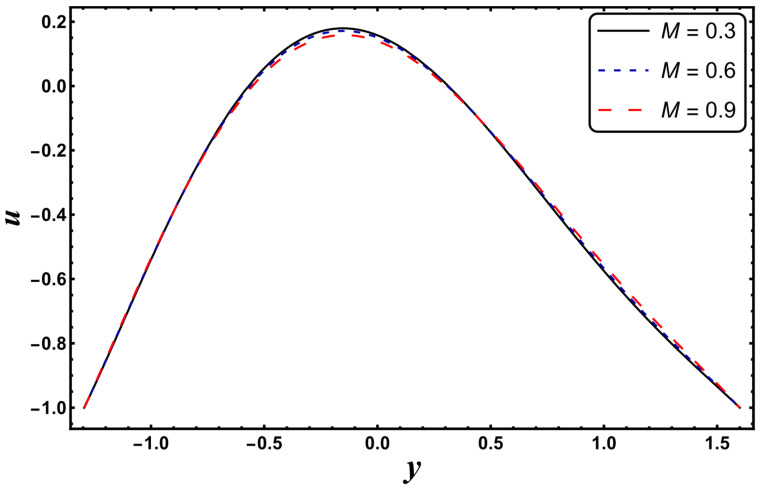
*u* for variation in *M*.

**Figure 29:**
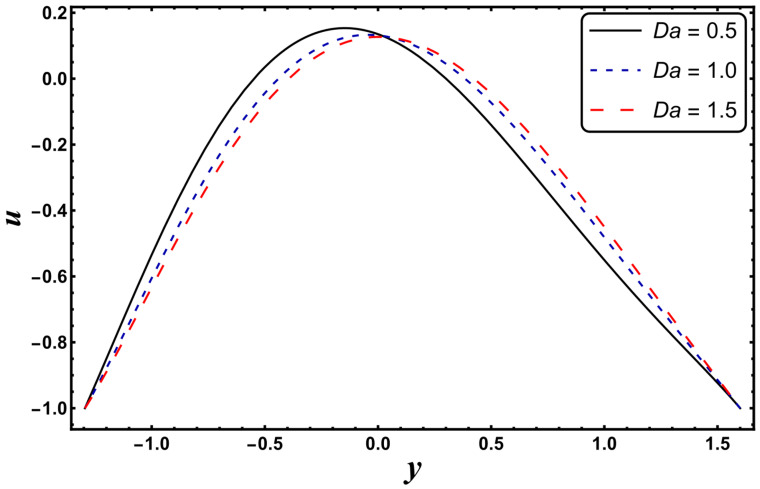
*u* for variation in *Da*.

**Figure 30:**
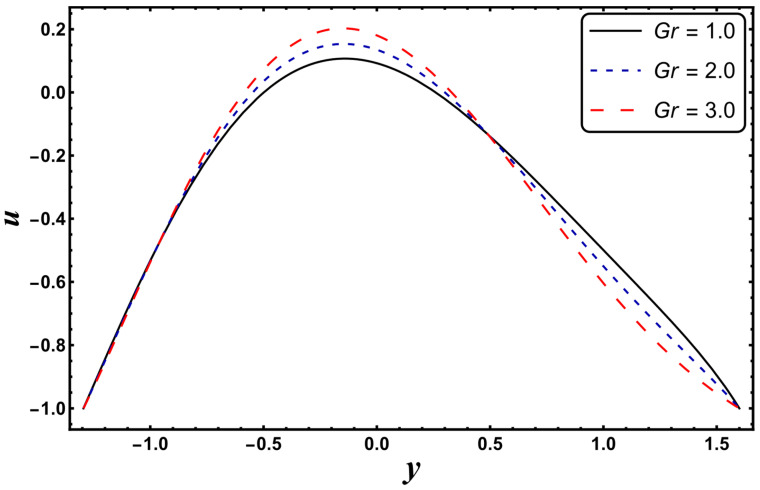
*u* for variation in *Gr*.

**Figure 31:**
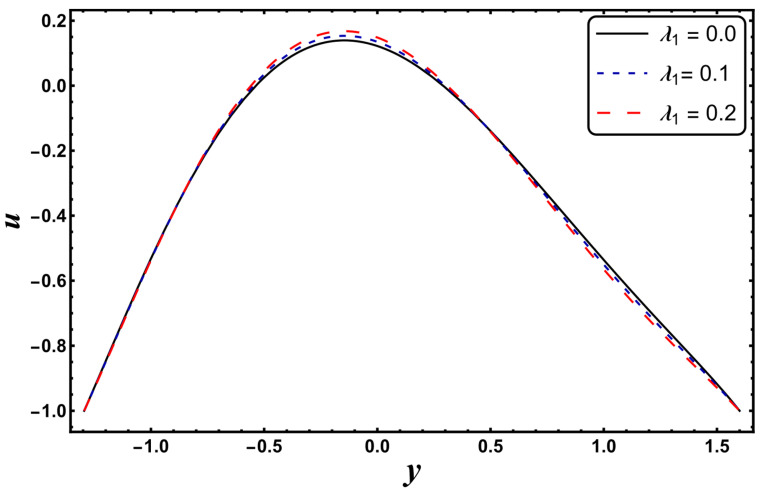
*u* for variation in *λ_1_*.

Figure 25, Figure 26, Figure 27, Figure 28, Figure 29, Figure 30 and Figure 31 elucidate the behavior of the velocity profile against *α*, *ω*, *U_hs_*, *Gr*, and *Da*. Figure 25 reveals that the velocity profile increases for larger values of variable viscosity, as increasing the variable viscosity parameter decreases the viscosity of the fluid and thus improves fluid movement. Figure 26 reveals that an improvement in the electroosmotic parameter increases the fluid flow. The electroosmotic parameter depends on the phenomenon of the electric double layer (ELD). Velocity lessens in the lower half of the channel but rises in the upper half of the channel, for the growing values of ‘*ω*’ when *U_hs_* = −1.0. Figure 27 delineates that velocity abates by enhancing ‘*U_hs_*’. For the auxiliary (assisting) electric field, velocity is highest, and it is lowest for the opposing electric field. Basically, ‘*U_hs_*’ is dependent on the electric field, and the electric field here primarily regulates the flow. ‘*U_hs_*’ is in direct proportion to the applied electric field. Thus, at positive values of ‘*U_hs_*’, it serves as an impeding force in the momentum equation, and at negative values it supports fluid flow. Figure 28 elucidates that velocity decreases for a larger ‘*M*’. A similar tendency was also seen by Zahid et al. [30]. It is known that the incidence of a magnetic field generates a Lorentz force (which is an opposing force in nature) that resists the buoyancy force and hence restricts the flow velocity in the channel. The external magnetic field clearly plays a major role in the control of peristaltic movement. The Darcy number has an increasing impact on the velocity profile near the channel center, and the reverse behavior is noted near channel walls (see Figure 29). However, a reverse phenomenon is encountered for the lower walls. Figure 30 delineates that velocity increases with increasing *Gr*. This is physically because with an augmentation in *Gr*, the buoyancy forces prevail over the viscous forces, which improves the fluid flow. A similar behavior was also encountered for the case of a higher ‘*λ*’ (see Figure 31).

### 4.7. Velocity at Channel Center

The numerical values of velocity at the channel center are shown in Table 2. The results from this table show that the values of velocity predicted using the kerosene were larger than those of the water and methanol. The gap between the values anticipated by three different fluids widens with an increase in the viscosity parameter. Velocity at the channel center increases for a higher *α*, *Gr*, and $\lambda_{1}$, and decreases for a larger *U_hs_*.

## 5. Concluding Remarks

This mathematical study analyzed the production of entropy and heat capacity of a kerosene-based working fluid moving by electroosmosis, as well as the propagation of peristaltic waves along the walls of an asymmetric channel. The problem was solved numerically in the computational software Mathematica. The obtained results improved the prospect of eliminating the causes of irreversibility. The main observations of this article are listed below:

- Temperature increased in the presence of electroosmotic and Joule heating parameters.
- A decrement in the heat transfer rate at the wall was encountered for the case of a higher Grashoff number.
- The radiation parameter was found to be very important for minimizing the generation of entropy. However, more irreversibility was created by mounting the values of the Joule heating parameter.
- The Bejan number decreased for the case of a higher Darcy’s number.
- Fluid velocity can be regulated by adjusting the magnetic field intensity and Helmholtz–Smoluchowski velocity.
- The nonlinear convection and viscosity parameter was an increasing function of velocity at the channel center. However, the reverse trend was noted for a higher Helmholtz–Smoluchowski velocity.

## Figures and Tables

**Table 1:** Numerical values of temperature at the channel center.

*α*	*Nr*	*Da*	*Gr*	*S*	Water	Methanol	Kerosene
**0.02**	1.0	0.5	2.0	0.5	0.9806	1.0013	1.3317
**0.04**					0.9793	0.9999	1.3262
**0.06**					0.9781	0.9984	1.3206
0.03	**0.5**				1.2938	1.3222	1.7818
	**1.0**				0.9800	1.0006	1.3289
	**1.5**				0.7930	0.8092	1.0655
		**0.5**			0.9800	1.0006	1.3289
		**1.0**			0.9387	0.9536	1.1912
		**1.5**			0.9239	0.9368	1.1418
			**0.0**		0.9720	0.9912	1.2874
			**1.0**		0.9756	0.9955	1.3061
			**2.0**		0.9800	1.0006	1.3289
				**0.0**	0.7184	0.7386	1.0597
				**1.0**	1.2419	1.2629	1.5994
				**2.0**	1.7668	1.7889	2.1448

**Table 2:** Numerical values of velocity at the channel center.

*α*	*U_hs_*	*Gr*	*λ_1_*	Water	Methanol	Kerosene
**0.02**	1.0	2.0	0.5	0.1076	0.1090	0.1328
**0.04**				0.1108	0.1123	0.1373
**0.06**				0.1141	0.1156	0.1420
0.03	**–0.5**			0.0949	0.0964	0.1200
	**0.0**			0.0808	0.0821	0.1052
	**0.5**			0.0666	0.0680	0.0905
		**0.0**		0.0508	0.0509	0.0520
		**1.0**		0.0798	0.0805	0.0924
		**2.0**		0.1092	0.1106	0.1350
			**0.0**	0.1092	0.1106	0.1350
			**0.1**	0.1156	0.1174	0.1483
			**0.2**	0.1220	0.1241	0.1591

## Data Availability

Not applicable.

## Nomenclature

($\vec{x}$, $\vec{y}$)	coordinates in wave frame (m)
($\vec{U}$, $\vec{V}$)	velocity components in lab frame (m s^−1^)
($\vec{u}$, $\vec{v}$)	velocity components in wave frame
*c*	speed of peristaltic wave (m/s)
$\vec{J}$	current density
$\vec{B}$	applied magnetic field (N A^−1^ m^−2^)
$\vec{E}$	applied electric field (N/C)
*C_f_*	specific heat of fluid
*μ_f_*	viscosity of fluid (Ns m^−2^)
*T*	dimensional temperature (K)
*δ*	wave number
$\sigma_{f}$	electric conductivity of fluid (S/m)
*g*	acceleration due to gravity (m s^−2^)
Φ	dimensional heat generation/absorption parameter (Wm^−2^K^−1^)
ε	dimensionless heat generation/absorption parameter
($\vec{X}$, $\vec{Y}$)	coordinates in lab frame
*P*	dimensional pressure
*T*_w_	temperature at channel wall
*p*	dimensionless pressure (N/m^2^)
*T*_0_	temperature of wall
*F*	dimensionless flow rate in wave frame
*θ*	dimensionless temperature
*ψ*	stream function
*η*	dimensionless flow rate in laboratory frame
*Pr*	Prandtl number
*K_f_*	thermal conductivity of fluid
*Re*	Reynolds number
*Br*	Brinkman number
*Ec*	Eckert number
*ρ_f_*	density of fluid (kg m^−3^)
*Gr*	Grashoff number
*M*	Hartman number

## References

[1] Aicher T., Martin H. (1997). New correlations for mixed turbulent natural and forced convection heat transfer in vertical tubes. Int. J. Heat Mass Transf..

[2] Bae Y.Y., Kim H.Y., Kang D.J. (2010). Forced and mixed convection heat transfer to supercritical CO2 vertically flowing in a uniformly heated circular tube. Exp. Therm. Fluid Sci..

[3] Kumar N., Doshi J.B., Vijayan P.K. (2011). Investigations on the role of mixed convection and wall friction factor in single-phase natural circulation loop dynamics. Ann. Nucl. Energy.

[4] Pretorius J.P., Kröger D.G. (2006). Critical evaluation of solar chimney power plant performance. Sol. Energy.

[5] Liu D., Gu H. (2017). Mixed convection heat transfer in a 5 × 5 rod bundles. Int. J. Heat Mass Transf..

[6] Jackson J.D. (2013). Fluid flow and convective heat transfer to fluids at supercritical pressure. Nucl. Eng. Des..

[7] Bae J.H., Yoo J.Y., Choi H., McEligot D.M. (2006). Effects of large density variation on strongly heated internal air flows. Phys. Fluids.

[8] Venugopal G., Balaji C., Venkateshan S.P. (2010). Experimental study of mixed convection heat transfer in a vertical duct filled with metallic porous structures. Int. J. Therm. Sci..

[9] Izadi M., Behzadmehr A., Shahmardan M.M. (2015). Effects of inclination angle on mixed convection heat transfer of a nanofluid in a square cavity. Int. J. Comput. Methods Eng. Sci. Mech..

[10] Majdi H.S., Abed A.M., Habeeb L.J. (2021). Mixed Convection Heat Transfer of CuO-H2O Nanofluid in a Triangular Lid-Driven Cavity with Circular Inner Body. J. Mech. Eng. Res. Dev..

[11] Latham T.W. (1966). Fluid Motions in a Peristaltic Pump. Ph.D. Thesis.

[12] Abdelsalam S.I., Bhatti M.M. (2018). The study of non-Newtonian nanofluid with hall and ion slip effects on peristaltically induced motion in a non-uniform channel. RSC Adv..

[13] Abdelsalam S.I., Bhatti M.M. (2018). The impact of impinging TiO_2_ nanoparticles in Prandtl nanofluid along with endoscopic and variable magnetic field effects on peristaltic blood flow. Multidiscip. Modeling Mater. Struct..

[14] Abdelsalam S.I., Vafai K. (2017). Combined effects of magnetic field and rheological properties on the peristaltic flow of a compressible fluid in a microfluidic channel. Eur. J. Mech. -B/Fluids.

[15] Eldesoky I.M. (2012). Influence of slip condition on peristaltic transport of a compressible Maxwell fluid through porous medium in a tube. Int. J. Appl. Math. Mech..

[16] Mekheimer K.S., Komy S.R., Abdelsalam S.I. (2013). Simultaneous effects of magnetic field and space porosity on compressible Maxwell fluid transport induced by a surface acoustic wave in a microchannel. Chin. Phys. B.

[17] Kothandapani M., Prakash J. (2015). Effect of radiation and magnetic field on peristaltic transport of nanofluids through a porous space in a tapered asymmetric channel. J. Magn. Magn. Mater..

[18] Abumandour R.M., Eldesoky I.M., Abdelwahab E.T. (2020). On the performance of peristaltic pumping for the MHD slip flow under the variation of elastic walls features. ERJ. Eng. Res. J..

[19] Rice C.L., Whitehead R. (1965). Electrokinetic flow in a narrow cylindrical capillary. J. Phys. Chem..

[20] Tang G.H., Li X.F., He Y.L., Tao W.Q. (2009). Electroosmotic flow of non-Newtonian fluid in microchannels. J. Non-Newton. Fluid Mech..

[21] Ramesh K., Prakash J. (2019). Thermal analysis for heat transfer enhancement in electroosmosis-modulated peristaltic transport of Sutterby nanofluids in a microfluidic vessel. J. Therm. Anal. Calorim..

[22] Akram J., Akbar N.S., Maraj E.N. (2020). A comparative study on the role of nanoparticle dispersion in electroosmosis regulated peristaltic flow of water. Alex. Eng. J..

[23] Akbar Y., Iqbal J., Hussain M., Khan H., Alotaibi H. (2022). Peristaltic transportation of Carreau–Yasuda magneto nanofluid embedded in a porous medium with heat and mass transfer. Waves Random Complex Media.

[24] Mahian O., Kianifar A., Sahin A.Z., Wongwises S. (2014). Entropy generation during Al2O3/water nanofluid flow in a solar collector: Effects of tube roughness, nanoparticle size, and different thermophysical models. Int. J. Heat Mass Transf..

[25] Mahian O., Mahmud S., Zeinali Heris S. (2012). Effect of uncertainties in physical properties on entropy generation between two rotating cylinders with nanofluids. J. Heat Transf..

[26] Ellahi R., Hassan M., Zeeshan A. (2015). Shape effects of nanosize particles in Cu–H2O nanofluid on entropy generation. Int. J. Heat Mass Transf..

[27] Sheikholeslami M., Ashorynejad H.R., Rana P. (2016). Lattice Boltzmann simulation of nanofluid heat transfer enhancement and entropy generation. J. Mol. Liq..

[28] Khan A., Khan I., Alkanhal T.A., Ali F., Khan D., Nisar K.S. (2019). Entropy generation in MHD conjugate flow with wall shear stress over an infinite plate: Exact analysis. Entropy.

[29] Akbar Y., Abbasi F.M. (2020). Impact of variable viscosity on peristaltic motion with entropy generation. Int. Commun. Heat Mass Transf..

[30] Zahid U.M., Akbar Y., Abbasi F.M. (2020). Entropy generation analysis for peristaltically driven flow of hybrid nanofluid. Chin. J. Phys..

[31] Tlau L., Ontela S. (2021). Effect of shape of nanoparticles on mixed convection nanofluid flow in a porous medium with variable permeability: Analysis of the second law of thermodynamics. Pramana.

[32] Ali Z., Qasim M., Ashraf M.U. (2021). Thermodynamic analysis of nonlinear convection in peristaltic flow. Int. Commun. Heat Mass Transf..

